# Familial adenomatous polyposis in an adolescent with coexisting schizophrenia: treatment strategies and implications

**DOI:** 10.1002/mgg3.114

**Published:** 2014-12-03

**Authors:** Luisa Gonzalez, Jose Alvarez, Erica Weinstein, Panagiota Korenis

**Affiliations:** 1 Bronx Lebanon Hospital Center, Albert Einstein College of MedicineBronx, New York; 2 Albert Einstein College of MedicineBronx, New York

**Keywords:** Adolescent, familial adenomatous polyposis, genetic, schizophrenia

## Abstract

Schizophrenia is associated with high mortality and morbidity. The etiology of schizophrenia remains unclear, studies implicate a multifactorial origin with genetic and environmental factors. The adenomatous polyposis coli (APC) gene has been associated with FAP (familial adenomatous polyposis), and studies have linked it to schizophrenia. However, there are few studies which examine the association between FAP and schizophrenia. Limited data exist regarding recommendations for genetic counseling of adolescents with comorbid psychiatric illness. A case of an adolescent with FAP who developed psychotic symptoms is presented. This case hopes to add to the literature about mental illness in those with FAP. A review of literature about the role of APC in schizophrenia as well as implications of genetic counseling on those who suffer with mental illness will be discussed.

## Introduction

Schizophrenia, a chronic and severe mental illness has been associated with high rates of mortality and morbidity leading to recurrent hospitalizations and increased risk of medical comorbidities. While the etiology of schizophrenia remains unclear, twin and adoption studies implicate a multifactorial origin with genetic factors playing a more dominant role than environmental factors (Levitt and Rodin 1992). One gene associated with schizophrenia has been the adenomatous polyposis coli (APC) gene; a tumor suppressor gene that increases one’s risk for cancer, specifically, colorectal cancer and familial adenomatous polyposis (FAP). Studies have confirmed an association with FAP and numerous medical comorbidities including: thyroid cancer, gastrointestinal tumors, medulloblastomas, and adrenal masses (Tibben 2011). Studies have also shown that FAP in one family member may lead to a high level of mental health problems in other members of the family, particularly adolescents including: oppositional defiant disorder, adjustment disorder, major depression, and anxiety disorder (Deng et al. 2013). However, there are few studies which examine the potential association between FAP and schizophrenia. Genetic counseling is recommended for those who have the familial pattern of FAP and may be at risk to inherit the disease, however, few recommendations and resources exist regarding counseling adolescents, specifically adolescents with comorbid psychiatric illness. Here, we present a case of a young woman with FAP inherited from her mother who developed psychotic symptoms including paranoia, thought disorder and hallucinations.

## Case Presentation

The identity of the patient has been concealed and replaced by a number to preserve patient confidentiality.

### History of present illness

The patient 1, an 18-year-old female, in 11th grade, domiciled, unemployed, living with her aunt who is also her legal guardian, with a history of schizophreniform disorder diagnosed 6 weeks prior, and FAP presented after her aunt activated EMS and the police due to psychosis. The patient’s mother deceased from colon cancer secondary to FAP also suffered from Schizophrenia. Patient 1 was subsequently admitted to the psychiatric hospital as she demonstrated paranoid ideations, hyperreligiosity, expansive affect and internal preoccupation, worsening over a number of days prior to admission.

### History of psychiatric and general medical illness

The patient was apparently well until 6 weeks prior to presentation when, over the course of a week, she demonstrated increasingly bizarre behavior. According to collateral history from an outside hospital, she stated she could only read from right to left on the page, like in Hebrew, and repeated “I am Jesus”, “Lord help me” multiple times. At that time, she was first hospitalized and stayed at an outside hospital for a 2-week period, where she was diagnosed with Schizophreniform disorder and was treated with Seroquel, an antipsychotic with mood stabilizing properties. However, after discharge, the patient only took one dose of the Seroquel before self-discontinuing due to the associated sedation she experienced. She did not attend her scheduled outpatient psychiatry intake appointment.

The patient was diagnosed with FAP at the age of 17, approximately 13 months prior to her first hospitalization, after genetic testing revealed a germline APC mutation R232X, resulting a premature truncation of the protein. Her mother, mother’s twin, and grandparent have also suffered from this familial colon cancer syndrome, as does her older brother. Her mother’s twin and older brother underwent prophylactic colectomy and patient 1 is currently being followed by the colorectal surgeon. The patient is likely to undergo colectomy in the next year, upon graduating from high school. The patient also has bilateral, subcentimeter thyroid nodules, which were identified on sonogram, and are being followed with an annual ultrasound exam.

### Family, development and social history

The patient was born to a single mother, the second of three children, her older brother shares the same father and the younger brother has a different father. She moved from Jamaica to New York City at age 3. However, she lived in Canada with her mother’s cousin for a number of years during her mother’s battle with cancer, returning at the age of 13 or 14 to New York. The mother died from colon cancer when the patient was 15 and her aunt became her legal guardian. The patient’s older brother, age 23, living alone, also possesses the APC gene mutation and underwent prophylactic surgery a number of years ago. Her younger brother, age 14, is without the APC mutation, and is in foster care as he has behavior and temper issues. Patient 1’s maternal aunt and mother’s identical twin has undergone multiple surgeries but is currently alive. Her mother, her mother’s twin, and a grandparent reportedly suffered from mental illness, likely schizophrenia. Her mother had been hospitalized a number of times in a psychiatric facility.

Pregnancy, birth, neonatal, and childhood history were reportedly unremarkable, except for a mild speech delay, for which she received therapy at age 3. The patient is currently in 11th grade, with just under an 80 average and is planning to complete high school and graduate next year, within the usual 4 years. Peers at school have bullied her in the past, with name calling about her appearance the most common subject of the verbal abuse. The patient and her aunt both deny any use of alcohol or illicit substance in the past or presently.

### Initial medical and psychiatric assessment

On initial arrival to our unit, patient 1 was acutely psychotic. She appeared young looking, with normal muscle tone and coordination and although she made appropriate eye contact at times, she did appear internally preoccupied. She stated her mood was “okay” but her affect was labile, with inappropriate, excessive laughter during the encounter. Her speech was impoverished and she whispered at times. Her thought process was tangential, and the content was with religious preoccupation, ideas of influence, paranoid delusions, and thought broadcasting. She believed her aunt, was poisoning her and that she represented Satan. She stated the police were cockroaches, produced from her Aunt, and were out to get her. The patient also endorsed thought broadcasting, stating she could communicate, via thoughts, with Venus and “Blue moon” in space. She denied suicidal or homicidal ideations. She was oriented to self and place but not oriented to time or purpose. She was forgetful with impaired abstracting ability and impaired concentration. Her insight was poor, as she stated she did not need medication because “I am the medication,” and her judgment was deemed to be impaired. Due to the acute onset of her symptoms, known polyposis of the colon, and her first presentation in our care, a first-break psychosis workup was done. The MRI of the head was unremarkable, as were the chest X-ray, EKG, laboratory work and drug screen.

### Hospital course

Patient 1 was admitted to the adult inpatient psychiatric unit. She was started on Haloperidol, an antipsychotic, 2 mg twice daily by mouth, and promptly demonstrated a response. Her psychotic symptoms began to abate and she stopped reporting perceptual disturbances. Her thought process became more coherent and organized. Her attention and concentration improved. After demonstrating a slight tremor and sedation after 10 days of treatment, the dose was adjusted to 2 mg daily at bedtime and she was discharged on this dose. The psychological tests demonstrated her intelligence was within the normal range. After remaining in the hospital for 13 days, she was discharged home and was able to return to school. She continues to follow up in the outpatient setting with a psychiatrist.

## Discussion

FAP is an inherited condition characterized by numerous polyps that form primarily in the epithelium of the large intestine but may also be found in the stomach and small intestine. Hundreds, sometimes thousands of polyps form with the onset of illness being around adolescence. If unrecognized and left untreated, this disorder leads to the development of colon cancer and accounts for 1% of all cases of colorectal cancer. By the age of 40, all patients will develop colorectal cancer and as a preventative measure, those diagnosed with FAP are recommended to undergo prophylactic colectomies. FAP is transmitted both spontaneously and in an autosomal dominant pattern, and has been linked to a mutation in the APC gene. APC is a tumor suppressor gene located on 5q21–22, a site reported to also be associated with schizophrenia (Attard 2007).

Since the abnormal gene that causes FAP is present in all of the body’s cells, other organs may develop growths. In more than 80% of patients with FAP, polyps form in the stomach and small intestine (Andrew and Jessen 2006). The polyps found in the duodenum are adenomas and may also become malignant. In fact, duodenal cancer is the second leading cause of cancer deaths in patients with FAP. The overall risk of duodenal cancer in FAP is about 4%. The other organs that form tumors include: skin, bones, eyes (congenital hypertrophy of the retinal pigment epithelium CHRPE), thyroid, and abdomen (Duncan and Savulescu 2005). Some patients with FAP are at increased risk for brain tumors, including cerebellar meduloblastomas.

### Genetic variants of FAP

While the polyps in FAP start out benign, malignant transformation into colon cancer occurs if left untreated. Three variants are known to exist, FAP and attenuated FAP (previously referred to as Hereditary Flat Adenoma Syndrome) are caused by APC gene defects. Autosomal recessive FAP or MYH-associated polyposis is caused by MUTYH gene defects (Hill et al. 2007). Of the three variants, FAP is the most severe and most common.

### FAP inheritance pattern

FAP can have multiple inheritance patterns and different genetic causes. When the condition is a result of the APC gene, it is inherited in an autosomal dominant pattern. Meaning that only one copy of the altered gene is sufficient to cause the disorder. The APC is a tumor suppressor gene and functions as a gatekeeper to prevent the development of tumors. APC regulates B-catenin, a protein that plays a crucial role in cell communication, signaling, growth, and controlled destruction (Durno and Gallinger 2006). When left unregulated, it gives rise to the production of hundreds, sometimes thousands of tumors in the intestinal wall which are predominantly benign, however, there is an increased risk of developing colorectal cancer. Studies suggest that there is a 7% risk by the age of 21, rising to 87% by the age of 45. At the age of 50, one’s risk has increased to 93% (Bussey 1982). The mutation in the APC gene does not trigger cancer but rather, it reduces the body’s ability to protect cells from becoming cancerous. In attenuated FAP, the APC gene is functional but slightly impaired. This form of FAP still has a 70% lifetime risk of cancer but typically presents with far fewer polyps (about 30) rather than the hundreds or thousands usually found in FAP. The age of onset is also later, presenting around the age of 40–70 years old rather than 30 years old like you would typically see in FAP. The third variant, autosomal recessive FAP is also milder and requires both parents to be carriers.

### Recommended course of treatment

Colectomy is recommended after adenomas emerge, specifically when there are more than 20 or 30 adenomas or multiple adenomas with advanced histology. Prophylactic surgery may be recommended before the age of 25 or upon detection if actively monitored. When the rectum is involved, the rectum and part or all the colon are removed (Chantada and Peeli 2005). The patient may require an ileostomy which is a permanent stoma where stool goes into a bag from the abdomen or they may have ileo-anal pouch reconstruction. The decision to remove the colon is based on the amount of polyps in the rectum as well as the family history. Because of these interventions, patients begin to have difficulty with medication absorption. Careful consideration has to be made when deciding medications, taking into consideration side effect profile. When the patient develops polyps in the duodenum or part of the stomach and undergoes resection they may have more difficulty absorbing medication. In mentally ill patients, specifically those who require psychotropic medication, there must be a mindful consideration of the type of medication prescribed and how it will be absorbed. The treating psychiatrist may consider using long-acting injectable medications rather than oral modalities.

### Counseling for FAP

In general, predictive genetic testing is offered to autonomous adults after counseling and consent. It has been well documented that genetic testing is recommended for adolescent and young adults with family history of FAP. Literature suggests it is appropriate to test at approximately the age of puberty to enable timely colonic surveillance and management of polyposis (Radhakrishnan and Bruc 2003).

Genetic testing has benefits as well as limitations. It can identify individuals at high or average risk, it leads to earlier screening and diagnosis, and guides toward earlier medical/surgical management. Some patients are very satisfied with their testing results, and testing helps them make decisions in reference to their medical as well as social issues (i.e., career choices, selecting a partner, child bearing, etc.) Challenges of genetic testing in young adults have been documented in the medical literature. There are concerns about predictive genetic testing of minors for several reasons. First, it removes the individual’s right to make an autonomous decision to be tested as an intellectually competent adult. Second, it denies them the right to confidentiality of results from parents and other family members. Third, identification of a minor as carrying a mutation has the potential for adverse emotional and psychological impact on the child (DudokdeWit and Tibben 1997). Some individuals respond negatively to these test results and experience increased anxiety due to concerns regarding the test not identifying all possible gene mutations. Moreover, others experience survivors guilt or guilt of having passed on the mutation to their child.

In FAP, counseling is recommended for all patients and their family members. Benefits include helping a person understand and cope with the anxiety and uncertainty of testing. Counselors can assist in the decision process regarding testing, screening, and interventions. They can help a person navigate and deal with the expected life style changes due to procedures or surgical interventions. Fear of discrimination in the work force or school due to physical limitations, as well as issues with body image and self esteem can be discussed and addressed by clinicians.

Counseling should be provided on an ongoing basis. The initial genetic testing and counseling may have been done at a time when the child/adolescent may not have had the ability to process the scope of the illness and its complications. It has been found that those who have had genetic counseling at an early age, recall only approximately two-thirds of the medical information presented (DudokdeWit and Tibben 1998).

Genetic counseling for individuals with chronic mental illness can present with even more challenges. The literature has shown that there is a need for a psychiatrist to refer their patients for genetic counseling and to provide awareness of this health service. For clinicians, it is especially important to establish rapport and encourage compliance, since they may be less likely to actively seek out referral or be unwilling to believe the information provided (Fernandez-Suarez and Cordero 2005). In general, support groups for FAP are available and indeed provide a venue for patients and family members to share information. However, few of such groups focus on assisting those with comorbid mental illness. Targeted research is required for the support and engagement of the chronically mentally ill patient with FAP.

### Recommendations for counseling a patient with mental illness and FAP

As clinicians, we should be mindful to not overload a psychiatric patient with excessive information during a period when that person is not stable or when they may have limited understanding of the illness and its implications. Ongoing screening for new symptoms as well as monitoring for substance use is recommended, particularly upon initiating genetic counseling.

Exploring patients feelings about living with the potential threat of developing cancer or adjustment to a possible premature death or loss of relatives affected with cancer is also recommended. In general, adherence to treatment for those with mental illness remains a struggle. When a patient presents with psychiatric and comorbid medical problems, it is imperative that the treating psychiatrist assess the patient’s adherence to recommended treatment, and have a good understanding of the patient’s community support system. Navigating the medical community is a challenge to most and may be a barrier to treatment compliance. To help with this, the treating psychiatrist may consider referrals to case management or other community resources. Further, it is also recommended that care be provided in a multidisciplinary approach whereby the medical team, genetic health professionals and psychiatric team work together to monitor the patient, and communicate regularly about medication changes and treatment recommendations.

## Conclusion

The association between FAP and schizophrenia is currently poorly understood and infrequently reported in the literature. Studies have linked APC to both FAP and schizophrenia. This case aims to add to the growing literature about the increased risk of mental illness in those diagnosed with FAP. We aim to bring to light the need for future investigations to better understand what role APC has in schizophrenia as well as the implications of genetic counseling on those who suffer with mental illness. Expanding knowledge and application of genetic counseling in psychiatry requires not only additional genetics training for psychiatrists, but also training in mental illness for medical genetics professionals.

## Conflict of Interest

None declared.
